# Development and validation of a machine learning model for post-PCI exercise intolerance in patients with coronary artery disease via electronic medical records

**DOI:** 10.3389/fpubh.2026.1751325

**Published:** 2026-02-10

**Authors:** LiHan Lin, Delong Li, YiPing Liu, GuoPeng Hu, Shiyi Lu, Zhiheng Li, Fanzheng Mu, Wei Zheng, Yongda Dong

**Affiliations:** 1 Provincial University Key Laboratory of Sport and Health Science, School of Physical Education and Sport Science, Fujian Normal University, Fuzhou, China; 2 College of Physical Education, Huaqiao University, Quanzhou, China; 3 Department of Cardiology, Quanzhou First Hospital Affiliated to Fujian Medical University, Quanzhou, China; 4 School of Physical Education and Health Care, Sanming University, Sanming, China

**Keywords:** coronary artery disease, electronic medical records, exercise intolerance, machine learning, percutaneous coronary intervention, predictive model

## Abstract

**Background:**

Exercise intolerance after percutaneous coronary intervention (PCI) is a common yet often overlooked condition in patients with coronary artery disease (CAD), associated with impaired cardiopulmonary recovery and poor prognosis. However, an accurate and easily applicable non-exercise-based model for predicting post-PCI exercise intolerance remains lacking. This study aimed to develop and validate such a model using electronic medical record (EMR) data.

**Methods:**

Between June 2020 and June 2024, clinical data were retrospectively collected from Quanzhou First Hospital. Forty-five variables were considered as candidate predictors, and seven machine learning algorithms were developed to estimate the risk of post-PCI exercise intolerance. Model performance was evaluated using the area under the receiver operating characteristic curve (AUC–ROC), area under the precision–recall curve (AUC–PRC), accuracy, sensitivity, specificity, positive predictive value (PPV), negative predictive value (NPV), and F1 score. Calibration and clinical utility were assessed via calibration plots, Brier score, Hosmer–Lemeshow (H–L) goodness-of-fit test, and decision curve analysis. Model interpretability was examined using Shapley additive explanations, and an interactive web-based calculator was deployed for clinical use.

**Results:**

A total of 575 patients were included, with an incidence of exercise intolerance of 22.0%. Eight key variables were selected: age, sex, BMI, smoking status, diabetes status, hemoglobin level, red blood cell count, and resting heart rate. The multilayer perceptron (MLP) model achieved the best performance (threshold = 0.30): an AUC–ROC of 0.911 (0.854–0.956), an AUC–PRC of 0.706 (0.548–0.846), an accuracy of 0.87, a sensitivity of 0.82, a specificity of 0.88, a PPV of 0.67, and an NPV of 0.94 (Brier = 0.108; H–L test *p* = 0.493).

**Conclusion:**

The proposed EMR-based model effectively identifies patients at high risk of post-PCI exercise intolerance, supporting early screening and targeted clinical interventions.

## 1 Introduction

Coronary artery disease (CAD) remains one of the leading causes of mortality worldwide (1). In China, its prevalence continues to rise with the aging population (2). As a principal treatment for CAD, PCI markedly improves clinical outcomes by rapidly restoring myocardial perfusion and reducing infarct size (3). However, patients with CAD generally exhibit poor exercise tolerance, which often further decreases following PCI, adversely affecting cardiopulmonary recovery and quality of life—an issue that is frequently overlooked in routine clinical practice (4–6).

Exercise tolerance (7, 8) refers to the maximal aerobic capacity an individual can sustain without developing pathological symptoms and is commonly assessed by peak oxygen uptake (VO_2_peak) measured during cardiopulmonary exercise testing (CPET) (9). According to the Weber classification, a VO_2_peak below 16 mL/kg/min is defined as exercise intolerance (10). Previous research has indicated that patients with coronary artery disease (CAD) who exhibit exercise intolerance are at substantially higher risk of coronary restenosis, major adverse cardiovascular events (MACEs), and all-cause mortality compared with those demonstrating preserved exercise capacity (11–14). Therefore, assessing exercise tolerance in post-PCI CAD patients serves as a direct indicator of prognosis and provides an essential basis for tailoring individualized cardiac rehabilitation prescriptions and predicting adverse cardiovascular outcomes. Despite its clinical value, the widespread application of cardiopulmonary exercise testing (CPET) remains constrained by significant financial costs, technical demands, and patient-specific limitations, including musculoskeletal impairments, severe comorbidities, or suboptimal adherence (15, 16). These limitations are particularly evident in China and other populous developing countries with unevenly distributed medical resources (17–19).

In recent years, machine learning (ML) has found widespread applications in healthcare (20–22). Among the essential data sources supporting these advances are electronic medical records (EMRs), which integrate comprehensive clinical data generated during hospitalization, including demographic characteristics, laboratory findings, vital signs, imaging results, and treatment details (23, 24). Given prior evidence that perioperative clinical indicators (e.g., hemoglobin, resting heart rate, blood pressure), medical history, and lifestyle factors are significantly associated with exercise tolerance in CAD patients undergoing PCI, EMR data provide a valuable resource for identifying potential predictive factors (25–28).

**Figure 1:**
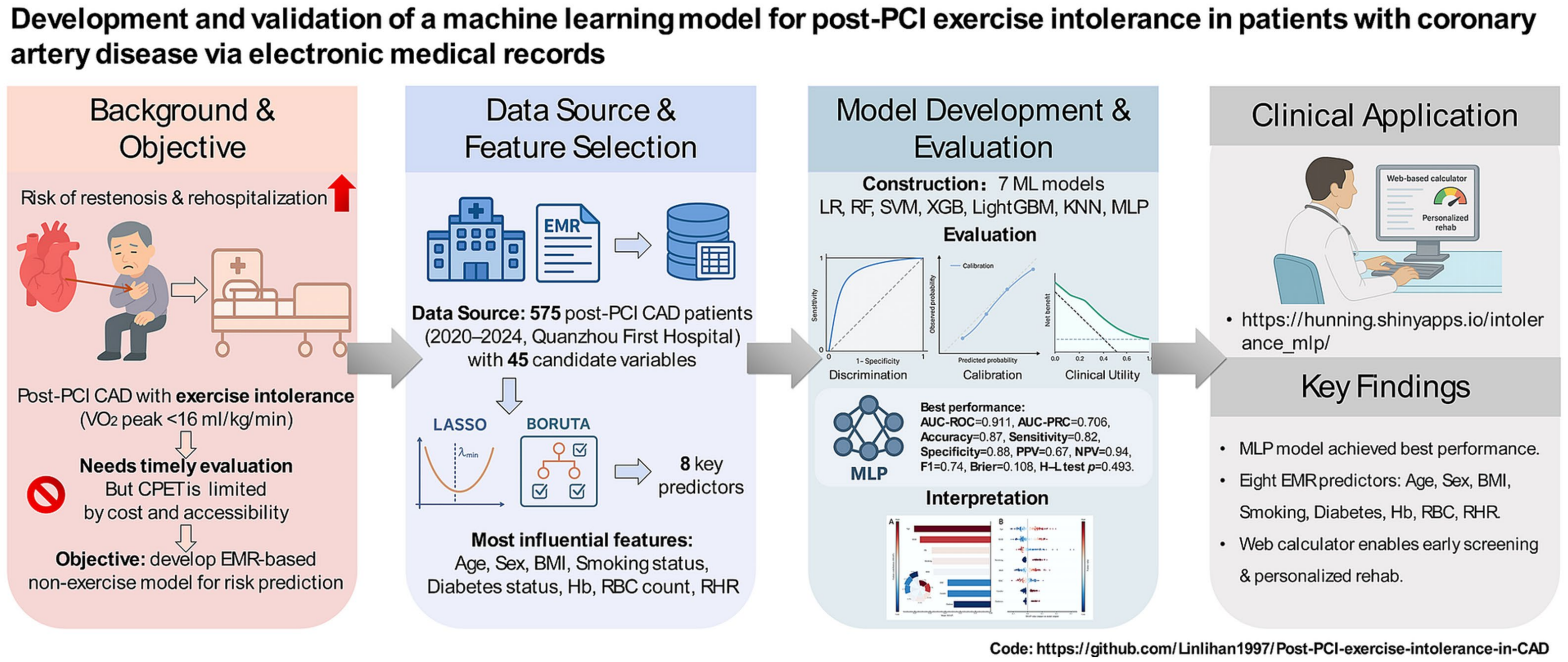
The graphical abstract for the study.

Therefore, this study aims to provide an effective alternative for patients unable to undergo CPET by (1) developing and validating an EMR-based predictive model for exercise intolerance after PCI in patients with CAD; (2) determining the relative importance of predictors through model interpretability analysis; and (3) deploying an online, clinically applicable prediction tool. Collectively, these efforts aim to support the prediction of exercise intolerance and the design of early intervention strategies in routine clinical practice. See Figure 1 for the graphical abstract of the study.

## 2 Methods

### 2.1 Participants

This retrospective analysis enrolled individuals with angiographically confirmed coronary artery disease (CAD) who received percutaneous coronary intervention (PCI) at Quanzhou First Hospital, Fujian Medical University, from June 2020 to June 2024. CAD diagnosis was determined in accordance with the American Heart Association (AHA) recommendations, characterized by ≥50% luminal narrowing in at least one major epicardial coronary vessel (29, 30), which defines CAD as ≥50% stenosis in at least one major epicardial coronary artery or its primary branches (left anterior descending, left circumflex, or right coronary artery). The investigators independently cross-checked the diagnostic and procedural data through comprehensive extraction from the hospital’s electronic medical records.

**Figure 2:**
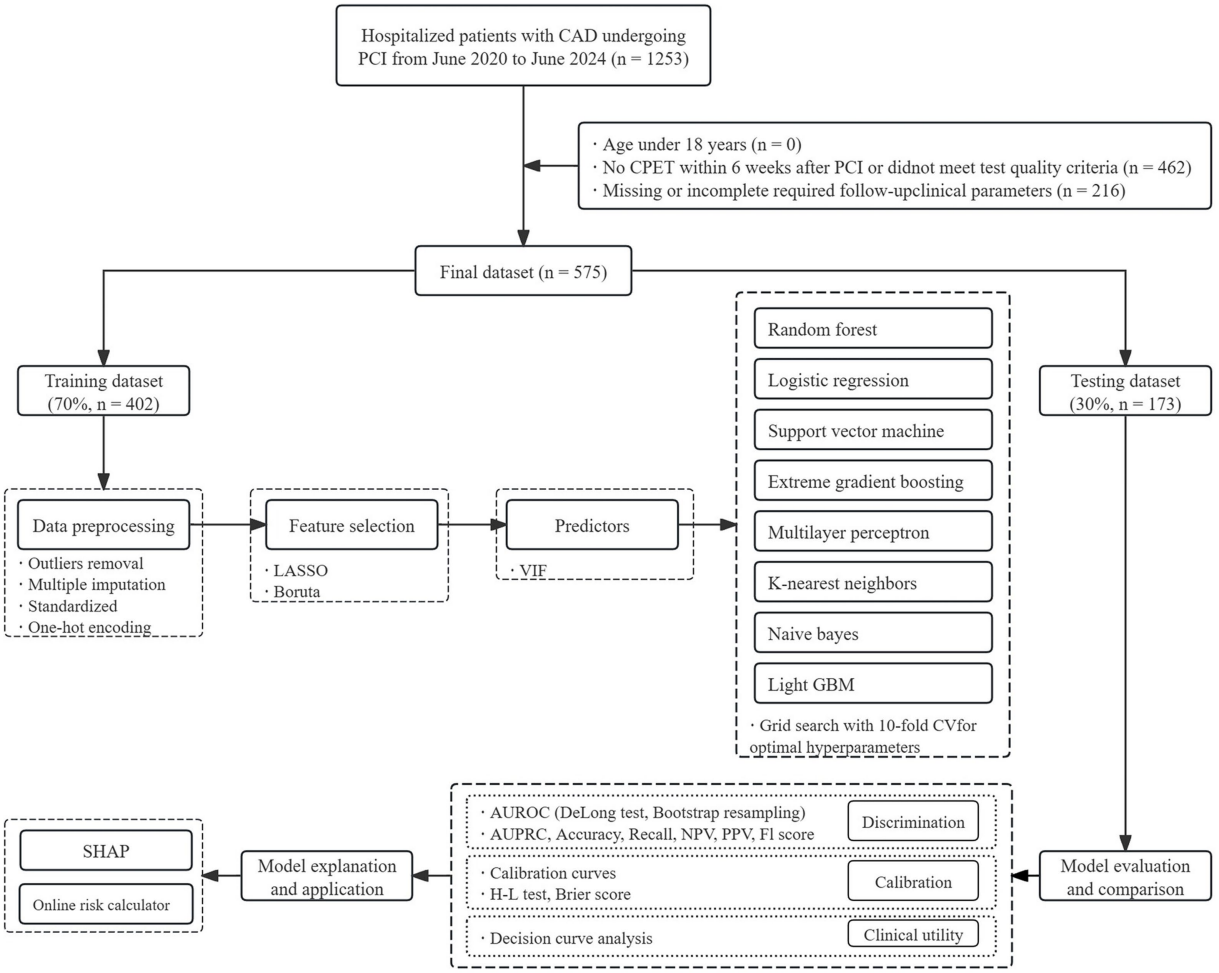
Flowchart of the study design.

Eligible participants met the following criteria: (1) age >18 years; (2) completion of a cardiopulmonary exercise test (CPET) within 6 weeks after PCI that satisfied the predefined quality requirements of the Chinese expert consensus on standardized clinical application (31); and (3) availability of complete echocardiographic, hematologic, and biochemical data at follow-up. Participants were excluded if they met any of the following conditions: (1) incomplete or missing follow-up data; (2) presence of critical cardiovascular or systemic diseases—such as recurrent post-PCI angina, cardiogenic shock, malignant arrhythmia, infective or inflammatory cardiac disorders (including endocarditis, myocarditis, or pericarditis), significant valvular lesions, malignancy, severe anemia, acute infection, advanced hepatic or renal dysfunction, or chronic pulmonary pathologies (e.g., moderate-to-severe COPD, pulmonary embolism, or interstitial fibrosis); (3) requirement for mechanical circulatory assistance during hospitalization (for instance, intra-aortic balloon pump or ventricular assist system); and (4) inability or refusal to complete cardiopulmonary exercise testing (CPET) due to orthopedic limitations, severe physical disability, or neuropsychiatric impairment. After exclusions, 575 post-PCI CAD cases remained eligible for analysis out of 1,253 initially screened records. A schematic overview of participant selection is provided in Figure 2.

This study was conducted in accordance with the principles outlined in the Declaration of Helsinki and received approval from the Ethics Committee of Quanzhou First Hospital, Fujian Medical University (Approval No. K052[2023]). The requirement for informed consent was waived because of the retrospective nature of the analysis.

### 2.2 Measurements

#### 2.2.1 Outcome definition

The primary outcome was postoperative exercise tolerance, defined via dichotomized VO_2_peak obtained from symptom-limited CPET. According to the Weber classification, a VO_2_peak <16 mL/kg/min was considered exercise intolerance, whereas a VO_2_peak ≥16 mL/kg/min was classified as exercise tolerance. In this analysis, VO_2_peak was assessed using a symptom-limited incremental cardiopulmonary exercise test conducted on a cycle ergometer (Vyntus CPX Metabolic Car, Vyaire Medical GmbH, Hoechberg, Germany). VO_2_peak was identified as the maximal oxygen consumption achieved during the final stage of exertion, calculated by averaging breath-by-breath data over the last 30 s of the exercise phase. A continuous ramp protocol was utilized, and system calibration was performed each day before participant testing to ensure measurement accuracy (32). Additional protocol details are provided in the Supplementary material S2 to ensure reproducibility.

#### 2.2.2 Candidate predictors

Guided by prior literature on determinants of exercise tolerance (33–36), and the availability of data in the EMRs, we prespecified 45 candidate predictors across three domains: demographics and medical history (11 variables), echocardiographic examination (24 variables), and laboratory testing (10 variables). In total, 45 candidate predictors were evaluated for their association with exercise intolerance. Detailed variable definitions, measurement procedures, and units are provided in the Supplementary material S2.

### 2.3 Machine learning models

#### 2.3.1 Data preprocessing and feature selection

Feature selection was performed in two stages. First, least absolute shrinkage and selection operator (LASSO) regression with 10-fold cross-validation was used to select predictors by shrinking irrelevant coefficients to zero. To assess robustness, the Boruta algorithm was applied to evaluate the relative importance of all the candidate variables. Multicollinearity was assessed via the variance inflation factor (VIF), with a VIF < 5 considered acceptable.

#### 2.3.2 Sample size

On the basis of our study cohort, the prevalence of exercise intolerance after PCI in patients with coronary artery disease was estimated to be 22.7%. Using this prevalence, the minimum required sample size for developing a reliable prediction model was calculated according to the criteria proposed by Riley et al. (37). Assuming a Cox–Snell *R*^2^ of 0.15, 10 candidate parameters, and a target shrinkage factor of 0.90, the required minimum sample size was estimated to be 549 participants (≈125 events). Our study sample exceeded this requirement.

#### 2.3.3 Model construction

This study constructs prediction models in strict accordance with the TRIPOD process (38). Seven machine learning algorithms have been developed for prediction: (LR) (39), random forest (RF) (40), support vector machine (SVM) (41), extreme gradient boosting (XGB) (42), light gradient boosting machine (LightGBM) (43), multilayer perceptron (MLP) (44), and k-nearest neighbors (KNN). The dataset was randomly divided into training (70%) and testing (30%) datasets. Hyperparameter optimization was conducted on the training set via stratified 10-fold cross-validation coupled with grid search to ensure balanced sampling across folds (45). The combination yielding the highest mean F1 score was selected as the optimal configuration. The independent testing dataset was then used for final model evaluation. Detailed descriptions of these machine learning models and the rationale for their selection are provided in Supplementary material S2.

#### 2.3.4 Model performance evaluation and interpretation

The discriminatory performance of the predictive models was evaluated via the area under the receiver operating characteristic curve (AUC–ROC), the area under the precision–recall curve (AUC–PRC), overall accuracy, recall, specificity, positive predictive value (PPV), negative predictive value (NPV), and F1 score. Model calibration was assessed via the Brier score, the Hosmer–Lemeshow goodness-of-fit test (H–L test, where *p* > 0.05 indicates no significant miscalibration), and visual inspection of calibration plots (reliability diagrams). A lower Brier score indicates better overall probabilistic accuracy, whereas the calibration plot reflects the agreement between the predicted and observed probabilities across risk deciles. Model performance was compared across multiple representative decision thresholds, including the default threshold (0.5) and the F1-optimal threshold. The optimal threshold for classification was determined on the testing dataset by maximizing the F1 score within the cross-validation framework. DeLong’s test was performed to assess the differences in the AUC–ROC.

Additionally, to facilitate clinical interpretation, we simulated the PPV and NPV of the best-performing model at each decision threshold under hypothetical outcome prevalences of 0.05, 0.10, 0.20, and 0.30, representing different clinical scenarios. To examine potential clinical utility, decision curve analysis (DCA) was performed across a range of threshold probabilities. To enhance interpretability, the optimal model was further analyzed via the Shapley additive explanations (SHAP) framework, which quantifies the relative contribution of each predictor to the model’s output at both the global and individual levels. An overview of the study design is provided in Figure 2.

### 2.4 Statistical analysis

Statistical analyses and data visualization were performed using SPSS 26.0, R 4.4.0, and JupyterLab 4.4.0 (Python 3.7). All tests were two-tailed, and *p* < 0.05 was considered statistically significant. Continuous variables conforming to a normal distribution are expressed as mean ± standard deviation and analyzed with the *t*-test, whereas skewed data are summarized as median (interquartile range) and compared with the Mann–Whitney *U* test. Categorical data are expressed as numbers (percentages) and evaluated using the chi-square test.

## 3 Results

### 3.1 Baseline characteristics

Among the 1,253 hospitalized patients with PCI-treated CAD, 575 participants were ultimately included in the study according to the inclusion and exclusion criteria. Among the 575 patients, 32.5% were female, with a median age of 64.0 years (IQR: 56.0–70.0), and 131 patients (22.0%) were classified as having exercise intolerance. For the overall cohort, the mean VO_2_peak was 19.29 ± 4.41 mL/kg/min, with values of 20.21 ± 4.54 mL/kg/min in males and 17.37 ± 3.41 mL/kg/min in females. The baseline characteristics of the study population are summarized in Table 1. Significant differences were observed in 18 baseline clinical and demographic variables between patients with and without exercise intolerance. The full cohort was further randomized at a 7:3 ratio into a training set (n = 402) and a testing set (n = 173). The baseline characteristics of the two sets, along with the number (percentage) of missing values for each variable, are summarized in Supplementary material S1. No significant differences were observed between the training and testing sets in most variables (*p* > 0.05), indicating comparability for ML model development. Descriptive metrics of major CPET parameters are provided in Supplementary material S2, while outcomes of the Shapiro–Wilk test assessing normality of continuous measures are detailed in Supplementary material S3.

**Table 1:** The characteristics of study participants.

Characteristics	Peak oxygen uptake, ml/kg/min			
	Exercise tolerance (*n* = 444)	Exercise intolerance (*n* = 131)	Statistic	*P*
Demographics and medical history				
Age, years	63.00 (55.00, 69.00)	70.00 (62.50, 75.00)	*Z* = −6.64	<0.001
Height, cm	165.00 (160.00, 170.00)	164.00 (157.50, 169.00)	*Z* = −3.44	<0.001
Weight, kg	67.00 (60.00, 75.12)	68.00 (60.00, 75.00)	*Z* = −0.49	0.624
BMI	24.62 ± 3.40	25.56 ± 3.53	*t* = −2.75	0.006
Gender			*χ*^2^ = 20.62	<0.001
Male	321 (72.30)	67 (51.15)		
Female	123 (27.70)	64 (48.85)		
Type of CAD			*χ*^2^ = 6.69	0.010
SA	286 (64.41)	68 (51.91)		
ACS	158 (35.59)	63 (48.09)		
Smoking			*χ*^2^ = 19.76	<0.001
No	325 (73.20)	69 (52.67)		
Yes	119 (26.80)	62 (47.33)		
Hypertension			*χ*^2^ = 14.09	<0.001
No	225 (50.68)	42 (32.06)		
Yes	219 (49.32)	89 (67.94)		
Hyperlipidemia			*χ*^2^ = 1.40	0.236
No	178 (40.09)	45 (34.35)		
Yes	266 (59.91)	86 (65.65)		
Diabetes			*χ*^2^ = 32.32	<0.001
No	354 (79.73)	72 (54.96)		
Yes	90 (20.27)	59 (45.04)		
CPET timing			*χ*^2^ = 0.66	0.720
<1 week	66 (14.86)	17 (12.98)		
1–3 weeks	247 (55.63)	71 (54.20)		
4–6 weeks	131 (29.50)	43 (32.82)		
Echocardiographic examination				
LVEDD, mm	47.00 (45.00, 50.00)	46.00 (45.00, 49.00)	*Z* = −1.37	0.171
LVESD, mm	29.00 (27.00, 32.00)	29.00 (27.00, 31.00)	*Z* = −0.44	0.660
IVSd, mm	9.00 (9.00, 10.00)	9.00 (9.00, 10.00)	*Z* = −0.42	0.673
LVPWd, mm	9.00 (8.00, 10.00)	9.00 (9.00, 10.00)	*Z* = −0.69	0.491
LA-ap, mm	34.00 (31.00, 37.00)	35.00 (31.00, 38.00)	*Z* = −1.35	0.175
AO-a, mm	21.00 (19.00, 22.00)	20.00 (19.00, 21.00)	*Z* = −0.76	0.444
AO-s, mm	33.00 (31.00, 36.00)	33.00 (31.00, 36.00)	*Z* = −0.17	0.862
AO-asc, mm	33.00 (30.00, 35.00)	33.00 (31.00, 36.00)	*Z* = −2.51	0.012
MPA, mm	22.00 (21.00, 23.00)	22.00 (21.00, 24.00)	*Z* = −1.95	0.051
EDV, mL	102.00 (92.00, 118.00)	97.00 (88.00, 113.00)	*Z* = −1.49	0.135
ESV, mL	34.00 (28.00, 41.00)	32.00 (27.50, 40.50)	*Z* = −0.45	0.650
EF, %	67.00 (62.00, 71.00)	66.00 (62.00, 70.50)	*Z* = −0.86	0.391
FS, %	38.00 (34.00, 41.00)	37.00 (34.00, 40.00)	*Z* = −0.86	0.392
SV, mL	68.00 (59.00, 77.00)	66.00 (58.00, 73.50)	*Z* = −1.41	0.159
CO, mL/min	4849.00 (4095.75, 5683.65)	4745.00 (4091.00, 5511.50)	*Z* = −0.65	0.517
CI, mL/min/m^2^	2833.50 (2437.75, 3222.25)	2819.00 (2417.00, 3261.60)	*Z* = −0.09	0.928
MV E, m/s	0.70 (0.60, 0.80)	0.70 (0.60, 0.80)	*Z* = −0.26	0.796
MV A, m/s	0.80 (0.70, 0.90)	0.90 (0.70, 1.00)	*Z* = −4.67	<0.001
E/A	0.86 (0.71, 1.12)	0.78 (0.64, 1.00)	*Z* = −3.75	<0.001
AV, m/s	1.20 (1.10, 1.40)	1.20 (1.10, 1.40)	*Z* = −1.04	0.296
PV, m/s	0.90 (0.80, 1.00)	0.90 (0.80, 1.00)	*Z* = −0.19	0.851
e’s, cm/s	7.00 (6.00, 8.00)	6.00 (5.00, 7.00)	*Z* = −3.88	<0.001
e’l, cm/s	9.00 (8.00, 11.00)	8.00 (7.00, 10.00)	*Z* = −2.70	0.007
E/e	9.00 (7.00, 10.00)	9.00 (7.50, 12.00)	*Z* = −2.73	0.006
Laboratory testing				
RHR, bpm	73.00 (68.00, 78.25)	81.00 (74.00, 90.00)	*Z* = −6.96	<0.001
RBC, 10^12/L^	4.73 (4.39, 5.01)	4.40 (4.07, 4.75)	*Z* = −6.11	<0.001
Hb, g/L	143.00 (134.00, 152.00)	132.00 (122.50, 142.50)	*Z* = −6.77	<0.001
HCT, %	41.75 (39.40, 44.40)	39.30 (36.25, 42.55)	*Z* = −5.71	<0.001
MCV, fL	88.70 (86.35, 91.32)	89.50 (86.55, 92.45)	*Z* = −0.97	0.334
TG, mmol/L	1.28 (0.94, 2.10)	1.45 (1.08, 2.04)	*Z* = −1.23	0.218
TC, mmol/L	4.80 (3.86, 5.78)	4.71 (3.62, 5.92)	*Z* = −0.21	0.836
LDL-C, mmol/L	2.94 (2.19, 3.73)	2.86 (1.94, 3.75)	*Z* = −1.02	0.307
HDL-C, mmol/L	1.29 (1.10, 1.58)	1.31 (1.09, 1.58)	*Z* = −0.16	0.873
CK, U/L	96.00 (67.00, 132.25)	89.00 (64.50, 119.00)	*Z* = −1.65	0.099

Exercise intolerance was defined as VO_2_peak <16 mL kg^−1^ min^−1^. Continuous variables are expressed as mean ± SD or median (IQR), as appropriate; categorical variables are shown as *n* (%). *t*, *t* test; *Z*, Mann–Whitney *U* test; *χ*^2^, Chi-square test.

### 3.2 Feature selection

**Figure 3:**
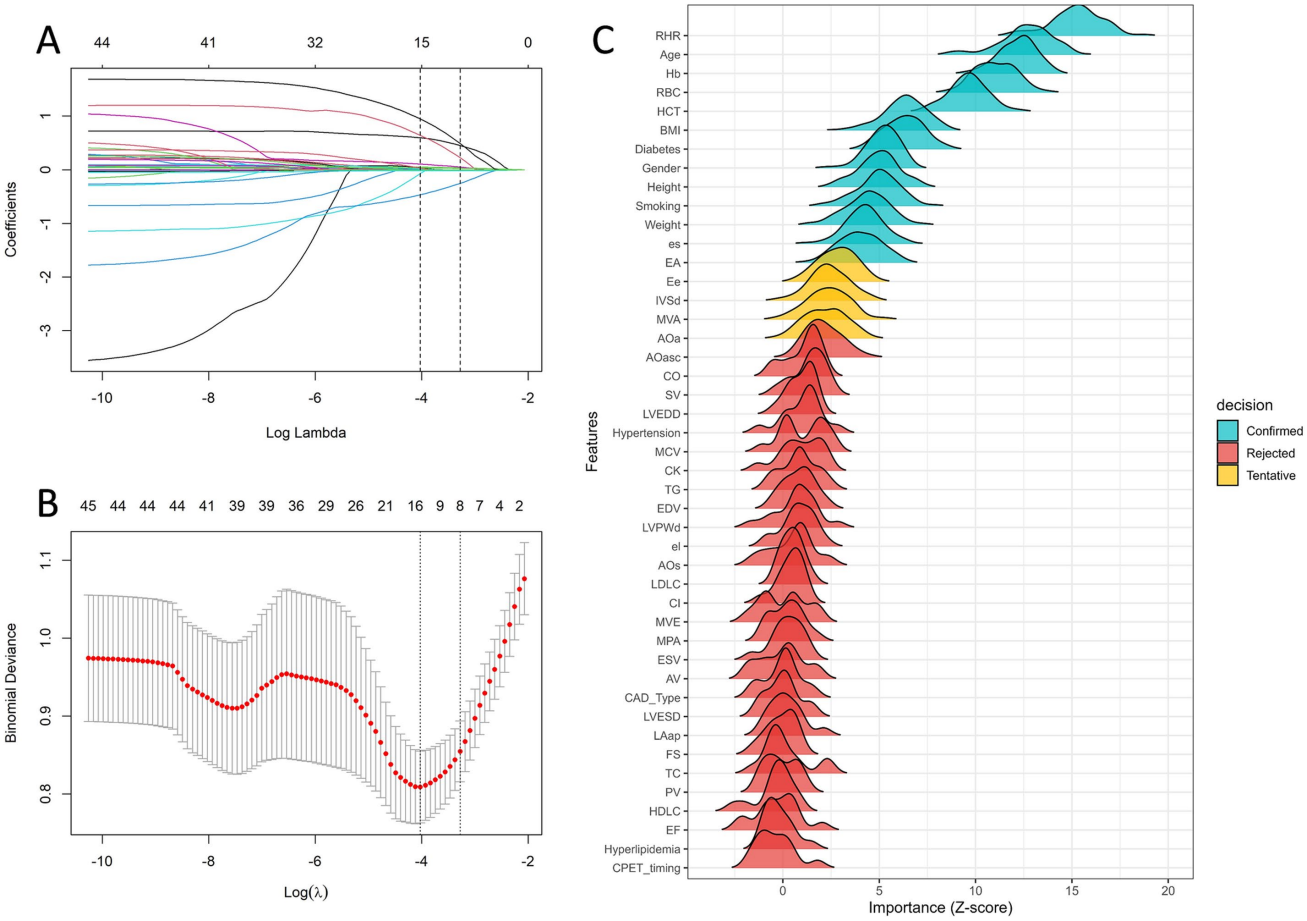
Feature variable selection via LASSO regression and the Boruta algorithm. **(A)** LASSO coefficient trajectories. Each curve represents a variable’s coefficient path along the sequence of log(*λ*). **(B)** Cross-validation for optimal *λ* selection. The left dashed line indicates *λ* corresponding to the minimum binomial deviance (*λ*min), and the right dashed line represents the one-standard-error criterion (*λ*1SE). **(C)** Feature importance according to the Boruta algorithm. The variables are categorized as “Confirmed” (teal), “Tentative” (yellow), or “Rejected” (red) according to their Z scores of importance.

To balance model interpretability and predictive performance, least absolute shrinkage and selection operator (LASSO) regression with 10-fold cross-validation was applied for preliminary feature selection, and the regularization parameter *λ* was determined at the minimum mean square error (λmin = 0.0178). At this threshold, a total of 15 nonzero coefficient variables were retained (Figures 3A,B). To further verify the robustness of feature selection, the Boruta algorithm was independently applied to evaluate the relative importance of all candidate variables (Figure 3C). Eight key predictors were identified as the most influential features, including age, sex, body mass index (BMI), smoking status, diabetes status, hemoglobin (Hb) level, red blood cell (RBC) count, and the resting heart rate (RHR). The multicollinearity assessment indicated no significant collinearity among the selected variables (Supplementary material S4).

### 3.3 Model construction and performance comparison

**Figure 4:**
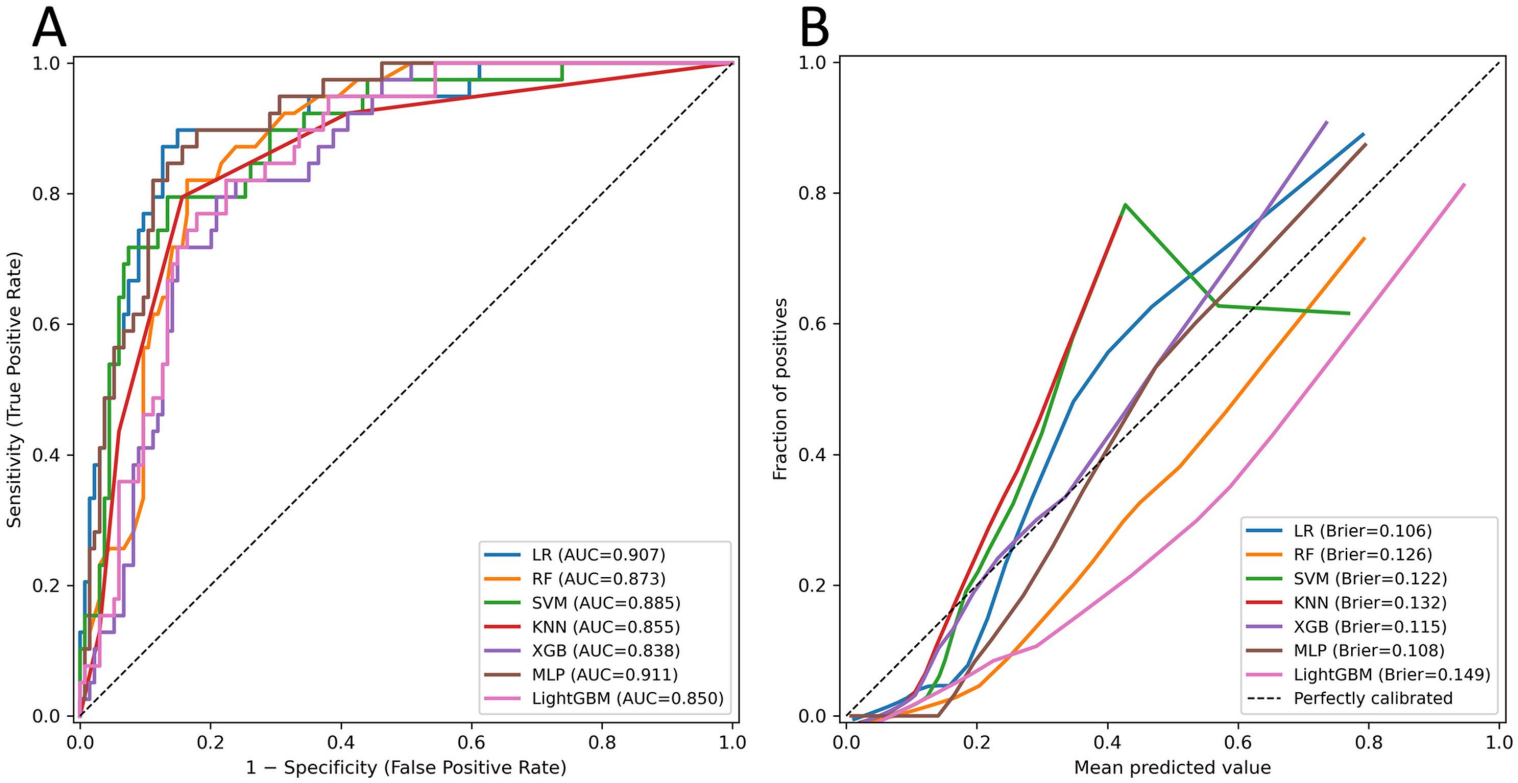
Performance comparison of seven machine learning models in the testing dataset: **(A)** ROC curves; **(B)** calibration curves.

Hyperparameter tuning was performed within predefined ranges (Supplementary material S5). The predictive performance of all the models is summarized in Table 2 for the testing set. The corresponding ROC and calibration curves are shown in Figure 4. All 7 machine learning models demonstrated good discriminative ability, with AUC–ROC values exceeding 0.85 across the testing dataset. Among them, the MLP 0.911 (0.854–0.956) and the LR 0.907 (0.850–0.954) achieved the highest discrimination. Pairwise DeLong tests revealed no statistically significant differences in the AUC–ROC curves between the models, indicating comparable discriminative capacity (Supplementary material S6).

**Table 2:** Performance metrics of different machine learning models in the testing dataset.

Models	Decision Label	Thresholds	AUC–ROC (95%)CI	AUC–PRC (95%)CI	Accuracy	Recall	Specificity	PPV	NPV	F1 score	H–L test *p*	Brier score
LR	Default	0.50	0.907 (0.850–0.954)	0.751 (0.590–0.867)	0.84	0.41	0.96	0.76	0.85	0.53	0.036	0.106
	F1-optimal	0.25			0.86	0.87	0.86	0.64	0.96	0.74		
RF	Default	0.50	0.873 (0.815–0.923)	0.586 (0.421–0.748)	0.77	0.28	0.92	0.50	0.81	0.36	0.053	0.126
	F1-optimal	0.25			0.80	0.82	0.79	0.53	0.94	0.65		
SVM	Default	0.50	0.885 (0.823–0.936)	0.685 (0.521–0.824)	0.77	0.28	0.92	0.50	0.81	0.36	0.038	0.122
	F1-optimal	0.30			0.80	0.82	0.79	0.53	0.94	0.65		
KNN	Default	0.50	0.855 (0.780–0.917)	0.606 (0.421–0.774)	0.78	0.05	0.99	0.50	0.78	0.09	0.003	0.132
	F1-optimal	0.25			0.74	0.74	0.74	0.45	0.91	0.56		
XGB	Default	0.50	0.838 (0.776–0.900)	0.507 (0.370–0.657)	0.79	0.46	0.88	0.53	0.85	0.49	0.359	0.115
	F1-optimal	0.21			0.81	0.72	0.84	0.56	0.91	0.63		
MLP	Default	**0.50**	**0.911(0.854–0.956)**	**0.706 (0.548–0.846)**	**0.82**	**0.28**	**0.97**	**0.73**	**0.82**	**0.41**	**0.493**	**0.108**
	F1-optimal	**0.30**			**0.87**	**0.82**	**0.88**	**0.67**	**0.94**	**0.74**		
Light GBM	Default	0.50	0.850 (0.788–0.907)	0.552 (0.393–0.704)	0.79	0.46	0.89	0.55	0.85	0.50	<0.001	0.149
	F1-optimal	0.24			0.80	0.77	0.81	0.54	0.92	0.63		

H–L test *P* > 0.05 indicates no significant miscalibration; Brier score < 0.25 suggests acceptable prediction accuracy. RF, random forest; LR, logistic regression; SVM, support vector machine; KNN, k-nearest neighbors; XGB, extreme gradient boosting; MLP, multilayer perceptron; Light GBM, light gradient boosting machine; AUC–ROC, area under the receiver operating characteristic curve; AUC–PRC, area under the precision–recall curve; PPV, positive predictive value; NPV, negative predictive value; H–L test, Hoslem–Lemeshow test. Bold indicates the best performance of the MLP model on the testing dataset.

Model calibration was evaluated via the Brier score, H–L test, and calibration plots. All the models demonstrated acceptable Brier scores (< 0.25), reflecting good overall prediction accuracy. However, LR, SVM, KNN (H–L test *p* < 0.05), and LightGBM (H–L test *p* < 0.001) showed significant miscalibration. Although the LR model exhibited strong discrimination and a low Brier score (0.106), its borderline H–L test result (*p* = 0.036) indicated mild miscalibration. Therefore, at the F1-optimal threshold, the MLP (threshold = 0.30) achieved the most balanced performance across discrimination, calibration, and clinical utility, with accuracy = 0.87, recall = 0.82, specificity = 0.88, PPV = 0.67, NPV = 0.94, and F1 = 0.74 (Brier = 0.108; H–L test *p* = 0.493). Overall, the MLP model demonstrated the most favorable trade-off between discrimination, calibration, and prediction stability and was selected as the optimal model for subsequent interpretability analysis.

### 3.4 Decision curve analysis

**Figure 5:**
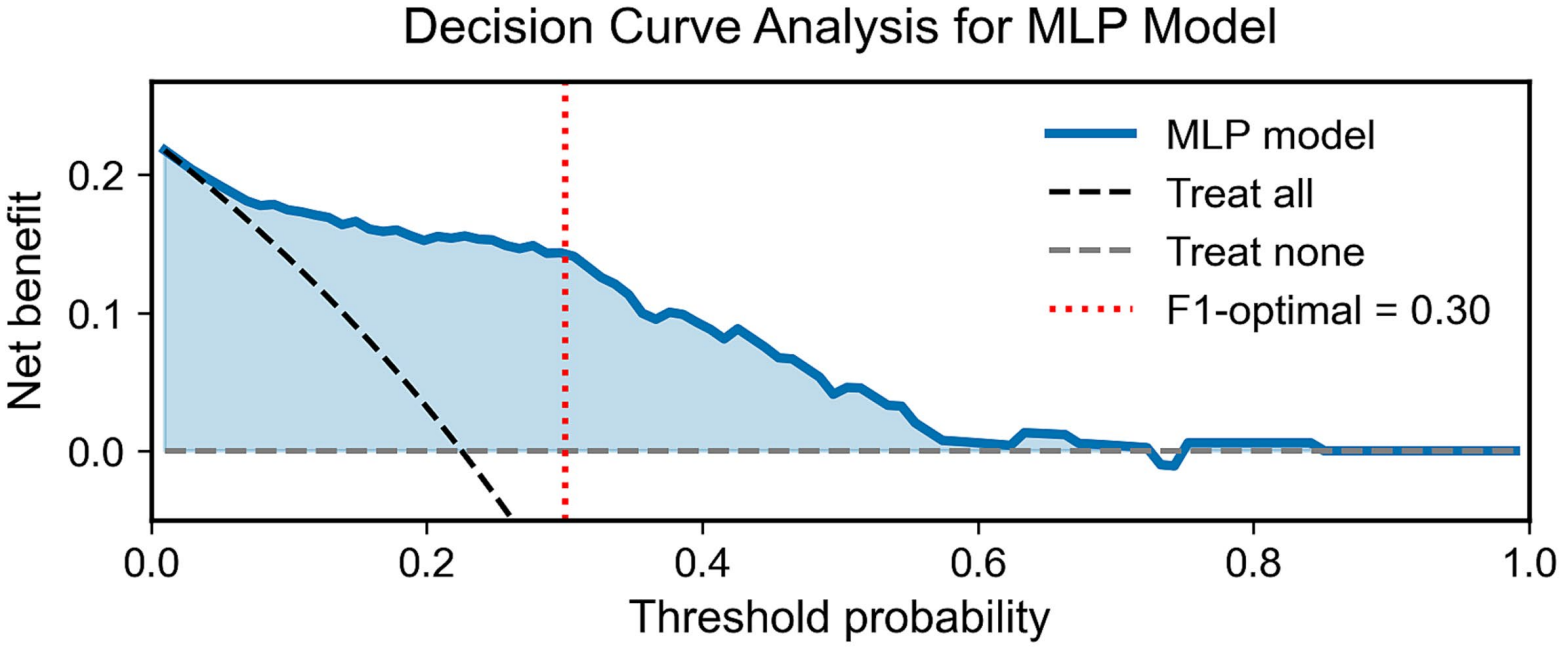
Decision curve analysis of the MLP model. The blue curve shows the net clinical benefit of the MLP model across threshold probabilities. The dashed black and gray lines represent the “treat all” and “treat none” strategies, respectively. The red dotted line indicates the F1-optimal threshold (0.30), where the model achieves the best balance between precision and recall.

As shown in Figure 5, the decision curve analysis demonstrated that the MLP model provided a clear clinical advantage over both the “treat-all” and “treat-none” strategies within threshold probabilities between approximately 0.05 and 0.30, reaching a maximum net benefit of around 0.22. The red dashed line indicates the F1-optimal threshold (0.30), showing that the model also yields substantial clinical net benefit at its statistically optimal cutoff. This advantage remained evident up to a threshold probability of roughly 0.70, suggesting that the MLP classifier offers clinically meaningful discrimination for identifying individuals with exercise intolerance across a wide range of decision thresholds.

### 3.5 Performance at representative decision thresholds

To aid clinical interpretability in identifying exercise intolerance, we simulated the PPV and NPV of the best-performing model (MLP) under varying assumed prevalence rates of poor exercise tolerance (Table 3). At the default threshold (0.50), the PPV improved from 0.33 at a 5% prevalence to 0.80 at a 30% prevalence, whereas the NPV decreased from 0.96 to 0.76. At the F1-optimal threshold (0.30), the model consistently achieves a high NPV across all prevalence levels (0.99–0.92) and increases the PPV with increasing prevalence (0.27–0.75). Compared with the default threshold, the F1-optimal setting offered greater sensitivity and comparable or superior PPVs, particularly under moderate-to-high prevalence conditions, suggesting better utility in real-world primary screening for exercise intolerance.

**Table 3:** Simulated PPVand NPV across different prevalence rates and classification thresholds.

Label (threshold)	Prevalence	Sim PPV	Sim NPV
Default (0.50)	0.05	0.33	0.96
	0.10	0.51	0.92
	0.20	0.70	0.84
	0.30	0.80	0.76
F1-optimal (0.30)	0.05	0.27	0.99
	0.10	0.43	0.98
	0.20	0.63	0.95
	0.30	0.75	0.92

### 3.6 Model interpretation

**Figure 6:**
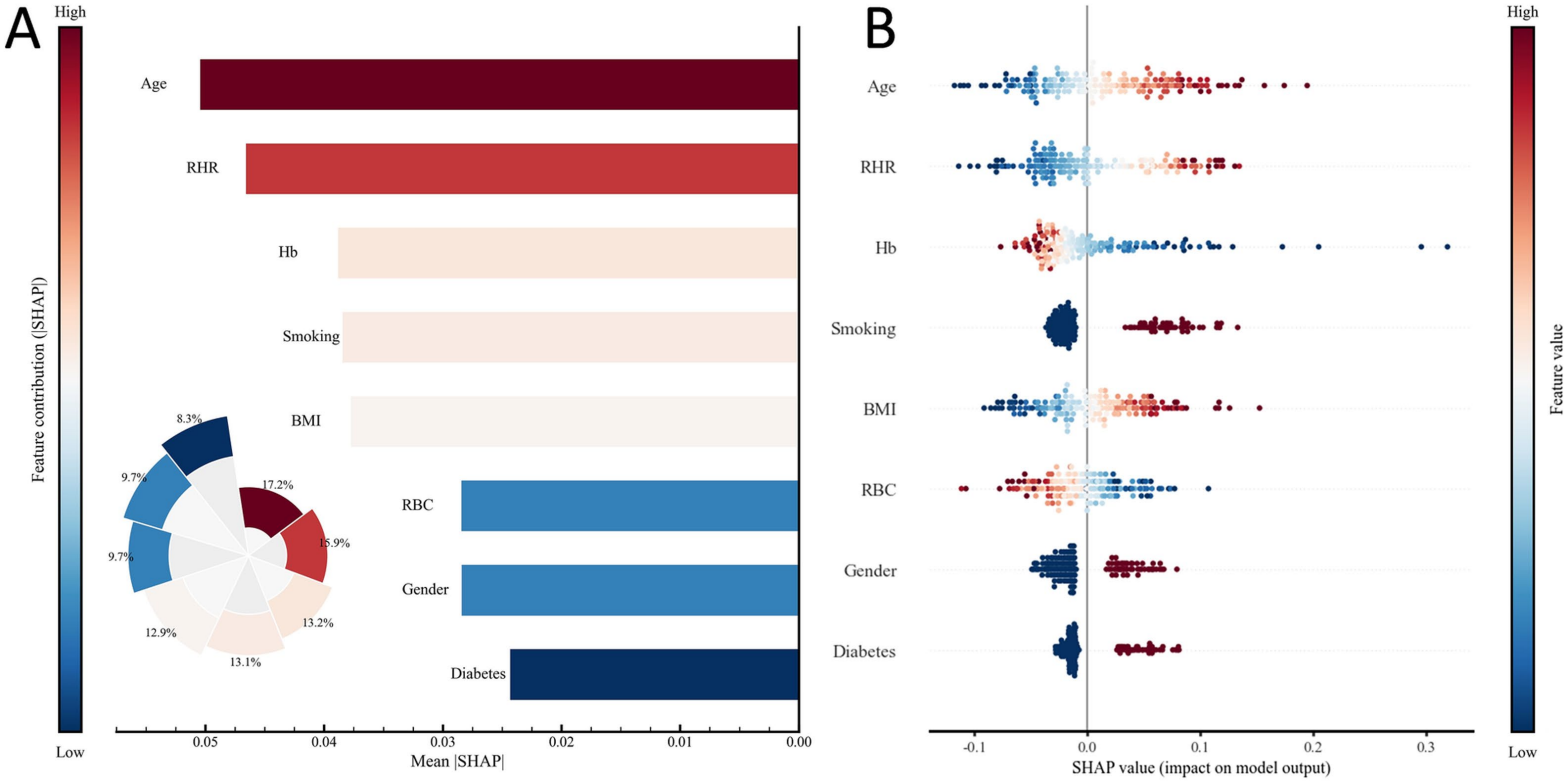
SHAP-based interpretation of the MLP model. **(A)** Feature importance ranking derived from the mean absolute SHAP values, reflecting each variable’s average contribution to the model output. **(B)** SHAP beeswarm visualization illustrating how feature magnitude and direction (color-coded) influence the model’s predicted probability for the positive outcome.

To elucidate the contribution of individual predictors to exercise intolerance, SHAP analysis was performed on the MLP model. Figure 6A illustrates the global importance of features on the basis of their mean absolute SHAP values. Age, RHR, and Hb were the strongest contributors to model output, followed by smoking, BMI, RBC count, sex, and diabetes, indicating their overall influence on distinguishing individuals with and without exercise intolerance. Figure 6B presents the SHAP summary plot, depicting both the direction and magnitude of each feature’s impact at the individual level. Higher age, elevated RHR, and lower Hb levels were associated with positive SHAP values, reflecting an increased predicted probability of exercise intolerance. Conversely, a lower BMI and nonsmoking status tended to yield negative SHAP values, suggesting a lower risk. The distribution of SHAP values also indicates nonlinear feature effects, where subtle variations in Hb or RBC count produce heterogeneous influences on the prediction outcome.

### 3.7 Online exercise intolerance risk calculator

**Figure 7:**
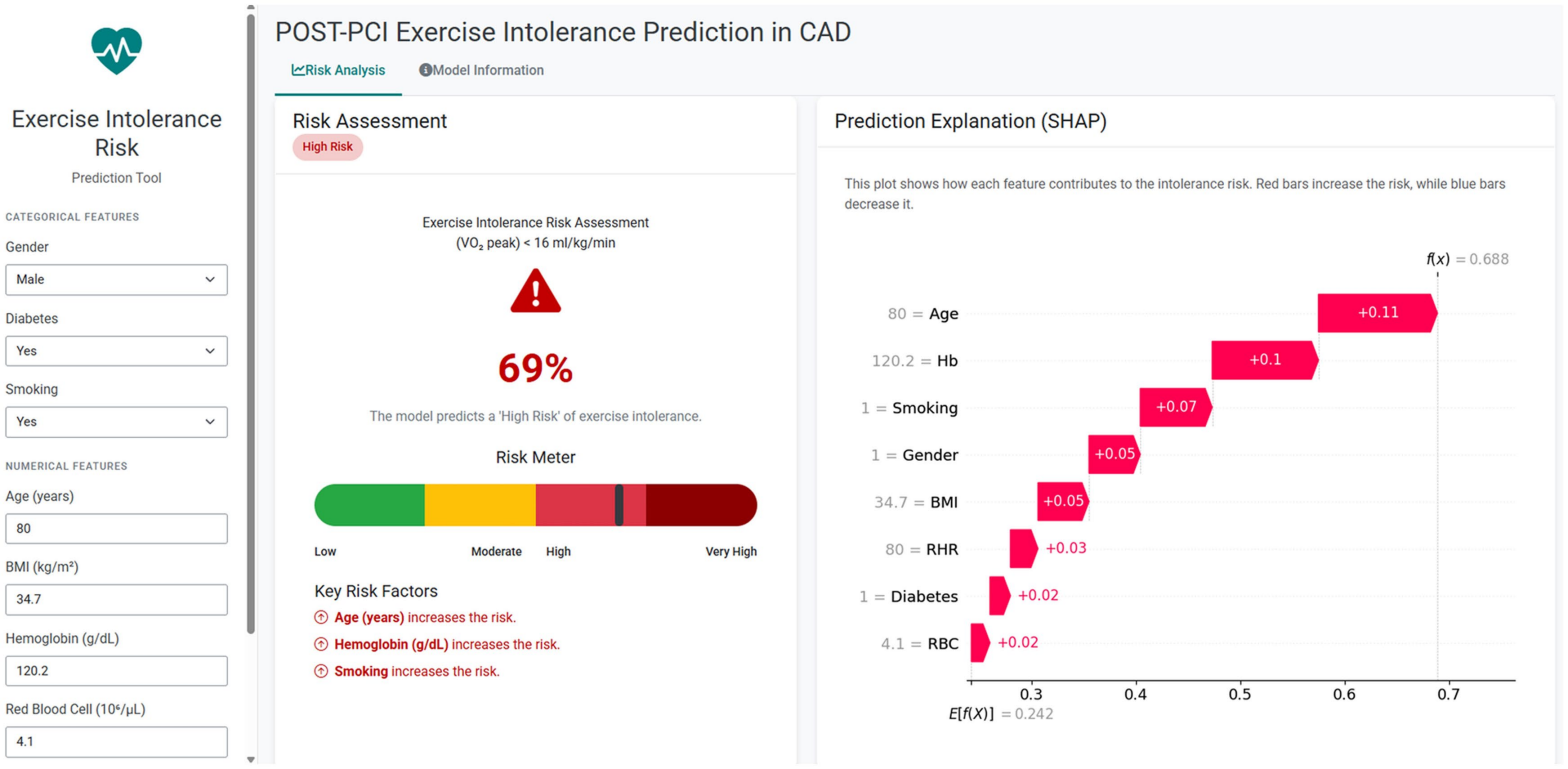
Operation page of the web risk calculator. The calculator allows users to input categorical and numerical features derived from EMRs to estimate the probability of exercise intolerance. The risk output is visualized through a color-coded risk meter and SHAP-based feature explanations, where red bars indicate positive contributions to risk. In this example, the predicted probability of exercise intolerance was 69%, with age, hemoglobin, and smoking being the strongest risk contributors.

To enhance clinical applicability, an interactive online risk calculator was developed on the basis of the optimized MLP model for the prediction of post-PCI exercise intolerance. The calculator is publicly accessible at https://hunning.shinyapps.io/intolerance_mlp. Clinicians can input eight routinely available parameters (age, sex, BMI, smoking status, diabetes status, Hb, RBC count, and RHR) to obtain an individualized probability of exercise intolerance and visualize the relative contribution of each predictor to the model output (Figure 7). The interface also provides classification results at F1-optimal thresholds, thereby supporting informed clinical decision-making and facilitating personalized exercise rehabilitation after PCI.

## 4 Discussion

In this study, we developed and validated seven ML models using the EMRs of 575 patients with CAD after PCI to predict the risk of exercise intolerance. Among the 45 candidate predictors, 8 key variables were identified as the most influential. Among all the models, the MLP demonstrated the best predictive performance, with age and RHR emerging as the strongest determinants. Importantly, we further developed an interactive online risk assessment tool and integrated SHAP values to enhance model interpretability, thereby underscoring the potential for the clinical translation of this study.

Compared with other ML algorithms, the MLP model showed superior discrimination (AUC–ROC = 0.911), good calibration (H–L test *p* = 0.493; Brier = 0.108), and consistent clinical net benefit. It achieved a high NPV (0.99–0.92) across prevalence levels of 0.05–0.30, with the PPV increasing from 0.27 to 0.75. This robust performance of the MLP model can be attributed to its ability to capture nonlinear relationships and complex feature interactions within a relatively small EMR dataset (46, 47). By leveraging eight carefully selected predictors, the MLP is likely because its nonlinear architecture captures complex interactions among the eight selected predictors while maintaining good generalizability in a modest-sized EMR dataset. Moreover, the nonlinear activation functions and multilayer architecture enable the MLP to model subtle physiological interdependencies—such as age-related declines in cardiopulmonary capacity or comorbidity-related metabolic variations—that linear models may overlook (48).

This study is the first to develop a machine learning–based predictive model for post-PCI exercise intolerance in patients with CAD via EMR data rather than exercise-testing data. Previous studies have relied primarily on either field-based assessments, such as the six-minute walk test (6MWT) or the incremental shuttle walk test (ISWT), or traditional statistical methods, such as logistic regression, to classify exercise tolerance on the basis of VO_2_peak thresholds, typically reporting AUC–ROC values between 0.76 and 0.85 (49–52). In contrast, our EMR-based MLP model achieved superior discrimination (AUC–ROC = 0.911), good calibration and clinical net benefit. Compared with traditional exercise-based assessments, nonexercise models can estimate exercise tolerance or cardiorespiratory fitness without the need for onsite testing, thereby reducing time, cost, and safety risks—particularly for patients with limited mobility. Furthermore, by leveraging routinely collected EMR variables, such models enable large-scale, automated screening and personalized risk prediction, which are difficult to achieve via conventional field or laboratory tests. Finally, we provide a comprehensive evaluation of model calibration and clinical utility and deploy an interactive web-based calculator to facilitate practical clinical implementation.

The developed model incorporated eight predictor variables—age, sex, BMI, smoking status, diabetes status, Hb level, RBC count, and RHR. Age emerged as the most influential feature, with the risk of exercise intolerance increasing progressively with advancing age. This finding is consistent with the Baltimore Longitudinal Study of Aging, which reported a decline in cardiorespiratory fitness of approximately 3–6% per decade from the third decade of life, accelerating to over 20% per decade after age 70, with a steeper decline observed in men after 40 years (53). These results suggest that the reduction in exercise tolerance among post-PCI patients is closely associated with age-related decreases in cardiovascular compliance, microvascular perfusion, and skeletal muscle mass. Consistently, age is also a key predictor in cardiorespiratory fitness models developed in general populations (54, 55). Therefore, older patients may benefit from targeted exercise interventions focused on muscle strengthening, microcirculation, and metabolic optimization to improve exercise tolerance and reduce recurrent cardiovascular risk. Furthermore, RHR was inversely associated with exercise tolerance, indicating that patients with higher RHR had a greater risk of exercise intolerance. This finding is consistent with the study by Yuko Kato et al. from the Cardiovascular Institute of Japan (56), which included 2,160 Asian patients with cardiovascular disease who underwent CPET and reported a negative trend between RHR and the VO_2_ peak (P trend < 0.0001). From a predictive modeling perspective, RHR is frequently included as an important feature in cardiorespiratory fitness models across healthy and chronic disease populations (57, 58). Previous studies have also demonstrated that postoperative cardiac rehabilitation improves peripheral muscle oxygen uptake and utilization efficiency, thereby reducing RHR and enhancing the VO_2_ peak (59, 60).

Among the laboratory indicators, Hb and RBC counts were identified as important predictors in the model. Post-PCI patients with lower Hb levels were more likely to experience exercise intolerance, which is consistent with previous findings in Asian populations (61). Studies have shown that anemia caused by reduced Hb is an independent predictor of mortality after PCI and is associated with a higher incidence of MACE within 30 days, elevated troponin and CK-MB levels, and prolonged hospitalization (62). Similarly, prior research has demonstrated that the RBC count is closely related to cardiac function and serves as a key biomarker in cardiovascular health management (63, 64). A large-scale Chinese cohort reported that, in heart failure patients, each 1.0 × 10^12^/L increase in RBC count reduced cardiovascular mortality risk by 28% in men and 43% in women, whereas low RBC levels were linked to a 1.5-fold higher risk of cardiovascular death (65). Taken together, these hematological findings highlight the importance of systemic oxygen-carrying capacity in determining postoperative exercise tolerance, whereas other demographic factors may also play crucial roles. Consistent with previous studies, sex was identified as a key predictor of exercise intolerance (66). In this study, the prevalence of exercise intolerance was significantly greater in female patients than in male patients (*p* < 0.001), which may be attributed to anatomical and physiological differences between the sexes. These include a smaller left ventricular size and lower stroke volume in women, reduced left ventricular diastolic compliance, and lower hemoglobin levels than in men (67–69). Feature importance analysis revealed that current smokers or those who had quit within the past year had a greater risk of exercise intolerance than nonsmokers did. This finding aligns with large-scale epidemiological studies showing that smokers with coronary artery disease have more severe coronary atherosclerosis, greater cardiac dysfunction, and a greater risk of myocardial infarction and coronary occlusion (70–72).

From a clinical standpoint, this study provides meaningful implications for improving the risk screening and management of exercise intolerance among patients with CAD after PCI. The predictive model offers clinicians an evidence-based and reproducible tool for assessing the risk of exercise intolerance. At present, the web-based calculator has not yet been formally evaluated within routine clinical workflows and should be considered a proof-of-concept implementation. The web-based calculator has the potential to be integrated into outpatient and inpatient workflows following appropriate field validation and usability assessment. In clinical settings, physicians can input readily available variables to generate individualized risk estimates. The interpretability of the model, which is enhanced through SHAP-based feature visualization, further assists clinicians in identifying modifiable risk drivers and tailoring personalized exercise prescriptions. For patients at intermediate risk, early lifestyle interventions—including structured cardiac rehabilitation, resistance training, and metabolic optimization—should be encouraged to improve exercise tolerance and reduce future cardiovascular risk. For high-risk patients, additional cardiopulmonary evaluations or multidisciplinary referrals may be warranted, with attention given to optimizing pharmacotherapy, managing anemia, and improving microcirculatory function. In future studies, the calculator may serve as a platform for prospective external validation by enabling the collection of real-world input data and outcome measures, allowing continuous evaluation of model performance. Over time, the developed tool may also facilitate patient education and shared decision-making, enabling low-cost, dynamic monitoring of functional recovery and response to rehabilitation. In this way, the management of post-PCI CAD can shift from reactive rehabilitation to proactive, risk-stratified exercise care, improving patient outcomes while optimizing resource allocation.

Despite its clinical relevance and practical implications, this study has several limitations. First, this was a single-center study, which may limit the generalizability of our findings to broader post-PCI populations with different clinical characteristics and care settings. Second, 462 patients were excluded due to unavailable or poor-quality CPET data, and an additional 216 were excluded because of missing or incomplete required follow-up parameters, which may introduce selection bias and limit the representativeness of the study sample. In addition, subgroup analyses by sex or age were not performed because of the relatively unbalanced sex distribution and the narrow age range of the cohort, which could lead to unstable subgroup-specific performance estimates. Third, although all participants completed CPET assessments within 6 weeks following PCI, slight variations in testing schedules across individuals might have introduced residual variability. Fourth, the absence of baseline exercise performance data before PCI limited our ability to distinguish procedure-related improvements from inherent inter-individual variability. Finally, habitual physical activity levels, psychological status, and the heterogeneous effects of pharmacological therapy that cannot be adequately captured by crude binary classification were not assessed, all of which may influence exercise tolerance. To address these limitations, future studies will focus on multicenter prospective validation, incorporating longitudinal follow-up and model-guided individualized interventions to evaluate their effectiveness in improving exercise tolerance and cardiopulmonary outcomes in patients with coronary artery disease after PCI.

## 5 Conclusion

In summary, an EMR-based model was developed to predict exercise intolerance in patients with CAD after PCI. The model demonstrated robust predictive performance and good interpretability, allowing early risk identification and targeted intervention. Older age, higher resting heart rate, lower hemoglobin levels, and smoking were key predictors, highlighting priority groups that warrant particular clinical attention.

## Data Availability

The datasets presented in this study can be found in online repositories. The names of the repository/repositories and accession number(s) can be found in the article/Supplementary material.
