# Supplementary material for: Development and validation of a machine learning model for post-PCI exercise intolerance in patients with coronary artery disease via electronic medical records
Source: Front Public Health. 2026 Feb 10;14:1751325. doi: 10.3389/fpubh.2026.1751325 (PMC12929426; doi:10.3389/fpubh.2026.1751325)
Supplement: Supplementary file 1 [file Table_1.docx]

**Development and validation of a machine learning model for post-PCI exercise intolerance in patients with coronary artery disease via electronic medical records**

**S2 Methods supplement**

**Standardized Cardiopulmonary Exercise Testing (CPET) Protocol**

This study followed the *Chinese expert consensus on standardized clinical application of cardiopulmonary exercise testing* (2022) and international practice guidelines. The CPET protocol was standardized as follows.

**1. Contraindications for CPET**

Contraindications were screened prior to testing, in accordance with the expert consensus

Absolute contraindications included uncontrolled acute coronary syndromes, acute heart failure, severe aortic stenosis or regurgitation, acute myocarditis or pericarditis, uncontrolled arrhythmias, acute pulmonary embolism, severe hypoxemia (resting SpO₂ <85%), acute deep venous thrombosis, or lack of informed consent.

Relative contraindications included known coronary stenosis ≥50% without revascularization, moderate valvular disease, hypertrophic cardiomyopathy, severe pulmonary hypertension, uncontrolled hypertension (SBP ≥180 mmHg or DBP ≥110 mmHg), resting HR ≥120 bpm, untreated endocrine or hematologic disorders (e.g., thyroid dysfunction, severe anemia, electrolyte imbalance), musculoskeletal injury, or mental disorders limiting exercise cooperation.

**2. Pre-test Preparation**

Environment: The CPET laboratory area was ≥20 m², equipped with ergometers, monitoring systems, and resuscitation equipment (defibrillator, oxygen supply, emergency medications). Room temperature was maintained at 20–22 °C, humidity at ~50%, with adequate lighting, ventilation, and privacy. A Borg rating of perceived exertion (RPE) scale was displayed on the wall.

Calibration: Gas analyzers and flow sensors were calibrated daily. O₂ and CO₂ analyzers were calibrated with reference gases before each test.

Participant preparation: Patients were required to be clinically stable, wear comfortable clothing and footwear, and fast for at least 2 h before testing. Caffeine, alcohol, and smoking were avoided within 2 h of testing. Patients were fitted with a face mask (or mouthpiece + nose clip) and instructed in the use of the Borg RPE scale.

Personnel preparation: Tests were supervised by trained physicians and technicians with certification in advanced life support.

**3. Test Procedure**

1. Application and physician evaluation: Each patient was assessed for medical history, current medication, smoking, daily activity level, and contraindications. Informed consent was obtained.

2. Resting period: A 3-minute seated rest with ECG, blood pressure, and respiratory parameters recorded.

3. Warm-up: A 3-minute unloaded pedaling at 0 W with cadence 55–65 rpm.

4. Ramp protocol: Workload was increased continuously at a rate of 10–30 W/min, individualized by the supervising physician according to health status, physical fitness, and anticipated exercise tolerance. Ramp rate selection followed consensus recommendations, aiming for volitional exhaustion in ~10–15 minutes. Frail patients were typically assigned 10–15 W/min increments, while fitter patients were assigned 20–30 W/min.

Formulae (per consensus):

- Predicted unloaded VO₂ (ml/min) = 150 + (6 × body weight [kg])
- Predicted peak VO₂ (ml/min) = (height [cm] − age [y]) × 20 (male) or ×14 (female)
- Recommended ramp rate (W/min) = (Predicted peak VO₂ − Predicted unloaded VO₂) / 100

5. Monitoring: ECG, blood pressure, oxygen saturation, respiratory gases, and patient symptoms were continuously recorded.

6. Recovery phase: After termination, patients pedaled unloaded for 2–3 minutes at 30–40 rpm, then were observed for 6–8 minutes until vital signs returned near baseline.

**4. Test Termination Criteria**

The test was terminated if:

- Patient reached 75–85% of age-predicted maximal HR or RER >1.05;
- Development of moderate to severe angina, severe dyspnea, dizziness, ataxia, pallor, or cyanosis;
- ST-segment depression ≥0.20 mV for ≥2 min or elevation ≥0.10 mV with arched upward configuration;
- Severe arrhythmias (ventricular tachycardia, frequent PVCs, AF with rapid ventricular response, AV block ≥ second degree);
- SBP drop ≥10 mmHg with increasing workload or SBP >220 mmHg / DBP >110 mmHg;
- Musculoskeletal fatigue or cramps leading to cadence decline;

Patient request to stop.

**5. Safety**

All tests were performed under direct supervision of an experienced cardiologist, with emergency equipment available.

**Overview of candidate predictors included in model development**

**1. Demographics and medical history**

A total of 12 demographic and clinical characteristics were collected: age, height, weight, gender, type of CAD, smoking status, comorbidities (hypertension, hyperlipidemia, diabetes), BMI, resting heart rate (RHR) , and CPET timing. VO _2_peak

CAD was classified as stable CAD or acute coronary syndrome (ACS), based on the primary diagnosis in medical records, consistent with previous studies [1, 2]. Smoking was defined per the WHO criteria: individuals with continuous or cumulative smoking for over six months were considered smokers [3]. Hypertension was defined as either: (1) systolic BP ≥140 mmHg and/or diastolic BP ≥90 mmHg without antihypertensive therapy [4]; or (2) self-reported physician diagnosis. Hyperlipidemia was defined as: (1) TC ≥6.22 mmol/L, LDL-C ≥4.14 mmol/L, TG ≥2.26 mmol/L, or HDL-C below threshold (men: <1.04 mmol/L; women: <1.30 mmol/L); or (2) self-reported diagnosis [5]. Diabetes was defined as: (1) FPG ≥7.0 mmol/L or HbA1c ≥6.5%; (2) positive oral glucose tolerance test (OGTT); or (3) self-reported diagnosis [6]. BMI is classified as normal (18.5–22.9 kg/m²), overweight (23.0–24.9 kg/m²), and obese (≥25.0 kg/m²), according to WHO recommendations for Asian populations [7]. RHR was measured by 24-hour resting ECG monitoring closest to the CPET date after PCI, using the PageWriter TC70 system (Philips, Amsterdam, Netherlands) [8]. CPET timing was categorized as <1 week, 1–3 weeks, and 4–6 weeks after PCI.

**2. Echocardiographic examination**

Transthoracic 2-dimensional, M-mode, and Doppler echocardiographic examinations were performed after PCI using digital ultrasound systems (EPIQ5C/EPIQ7C, Philips, Amsterdam, Netherlands; Vivid-7, GE Healthcare, Chicago, IL) equipped with a phased array transducer. A total of 24 parameters were assessed, including left ventricular end-diastolic diameter (LVEDD), left ventricular end-systolic diameter (LVESD), interventricular septal diastolic thickness (IVSd), left ventricular posterior wall diastolic thickness (LVPWd), left atrial anteroposterior diameter (LA-ap), aortic annulus diameter (AO-a), aortic sinus diameter (AO-s), proximal ascending aorta diameter (AO-asc), main pulmonary artery diameter (MPA), left ventricular end-diastolic volume (EDV), left ventricular end-systolic volume (ESV), ejection fraction (EF), fractional shortening (FS), stroke volume (SV), cardiac output (CO), cardiac index (CI), early mitral inflow velocity (MV E), late mitral inflow velocity (MV A), E/A ratio, aortic valve flow velocity (AV), pulmonary artery flow velocity (PV), early diastolic septal mitral annulus velocity (e’s), early diastolic lateral mitral annulus velocity (e’l), and E/e’ ratio. These echocardiographic indices provide a comprehensive assessment of cardiac structure and function [9].

**3. Laboratory testing**

All laboratory measurements were performed at the central laboratory of the First Hospital of Quanzhou. Complete blood count parameters, including red blood cell (RBC) count, hemoglobin (Hb), hematocrit (HCT), and mean corpuscular volume (MCV), were measured using automated analyzers (XW-100, Sysmex, Kobe, Japan; DxH 560AL, Beckman Coulter, Brea, CA, USA). Serum triglycerides (TG), total cholesterol (TC), low-density lipoprotein cholesterol (LDL-C), high-density lipoprotein cholesterol (HDL-C), fasting plasma glucose (FPG), and creatine kinase (CK) were determined using automated biochemical analyzers (LABOSPECT-006/7180, Hitachi, Tokyo, Japan). For each patient, the results were obtained from the laboratory testing performed closest to the CPET following PCI.

**Development and validation of a machine-learning model for post-PCI exercise intolerance in patients with coronary artery disease using electronic medical records**

Eight supervised machine learning algorithms were implemented to predict post-PCI exercise intolerance in patients with coronary artery disease (CAD). Each algorithm represents a different methodological paradigm, allowing a comprehensive comparison of linear, nonlinear, ensemble, and probabilistic approaches.

**1. Logistic Regression (LR)**

Logistic regression serves as a classical baseline model, which estimates the probability of a binary outcome through a logit link function. It assumes a linear relationship between independent variables and the log-odds of the dependent variable. Despite its simplicity, LR provides interpretable coefficients that quantify the direction and strength of associations between predictors and outcomes, thus helping to identify clinically meaningful risk factors for exercise intolerance.

**2. Random Forest (RF)**

Random forest is an ensemble learning method that constructs multiple decision trees using bootstrapped samples and randomly selected features at each split. The final prediction is obtained by aggregating (majority voting or averaging) across trees, reducing variance and improving generalization. RF can automatically capture nonlinear relationships and interactions among variables and is robust to multicollinearity and outliers, which are common in real-world electronic medical records (EMR) data.

**3. Support Vector Machine (SVM)**

SVM constructs an optimal separating hyperplane in a high-dimensional feature space to maximize the margin between classes. By employing kernel functions (e.g., radial basis function), SVM can model complex nonlinear decision boundaries. It performs well on high-dimensional data and is less prone to overfitting, making it suitable for clinical datasets with correlated or non-Gaussian features.

**4. Extreme Gradient Boosting (XGB)**

XGB is a scalable and efficient implementation of gradient boosting decision trees. It sequentially builds weak learners that correct errors of prior models through gradient descent optimization, incorporating regularization to prevent overfitting. XGB often achieves state-of-the-art predictive performance in structured medical datasets and allows feature importance estimation, facilitating model interpretability in clinical contexts.

**5. Light Gradient Boosting Machine (LightGBM)**

LightGBM is a tree-based gradient boosting framework that improves computational efficiency via histogram-based decision tree learning and leaf-wise growth strategies. It handles large-scale, high-dimensional EMR data efficiently and supports categorical features directly. Compared with XGB, LightGBM provides faster training speed and lower memory usage while maintaining competitive accuracy.

**6. Multilayer Perceptron (MLP)**

MLP is a feed-forward artificial neural network composed of interconnected layers of nodes. It captures complex nonlinear mappings through multiple layers of nonlinear transformations. With appropriate regularization and hyperparameter tuning, MLP can approximate any continuous function, enabling it to model intricate interactions between clinical variables and exercise capacity outcomes.

**7. K-Nearest Neighbors (KNN)**

KNN is a non-parametric algorithm that classifies samples based on the majority label among their nearest neighbors in the feature space. It relies on distance metrics to measure similarity between patients, providing an intuitive, instance-based prediction approach. Although sensitive to feature scaling, KNN can capture local structure in the data and is useful as a complementary benchmark to parametric models.

**8. Advantages of the machine-learning framework in this study**

Integration of multimodal clinical information: EMR data include heterogeneous variables (demographics, hematologic indices, comorbidities, and physiological measures). Machine learning enables the integration of such diverse features without rigid distributional assumptions.

Nonlinear and high-dimensional modeling: Algorithms such as RF, XGB, LightGBM, and MLP can model nonlinear, hierarchical, and interactive relationships between predictors and outcomes, which traditional regression often fails to capture.

Improved predictive performance and generalizability: Ensemble and boosting methods reduce overfitting by aggregating multiple weak learners and employing internal regularization, enhancing model stability across subpopulations (e.g., age or sex strata).

Explainability and clinical interpretability: Techniques such as feature importance ranking, SHAP (Shapley Additive Explanations), and partial dependence plots allow visualization of how each variable contributes to the prediction, bridging the gap between complex models and clinical insight.

Data-driven discovery and personalized rehabilitation: By leveraging large-scale EMR data, these algorithms identify subtle patterns associated with exercise intolerance, supporting individualized post-PCI exercise prescription and stratified cardiac rehabilitation strategies.

Robustness to noise and missingness: Tree-based and probabilistic algorithms tolerate imperfect or incomplete EMR inputs better than conventional statistical models, improving real-world applicability in clinical practice.

**Abbreviations**

ACS Acute Coronary Syndrome

AO-a Aortic Annulus Diameter

AO-asc Proximal Ascending Aorta Diameter

AO-s Aortic Sinus Diameter

AT Anaerobic Threshold

AV Aortic Valve Blood Flow Velocity

BMI Body Mass Index

CAD Coronary artery disease

CI Cardiac index; Confidence interval

CK Creatine Kinase

CO Cardiac Output

CPET Cardiopulmonary Exercise Testing

E/A Mitral Valve E/A Ratio

E/e' Early Diastolic to Late Diastolic Wave Ratio

EDV End-Diastolic Volume

EF Ejection Fraction

e'l Mitral Lateral Annulus Early Diastolic Velocity

e's Mitral Septal Annulus Early Diastolic Velocity

ESV End-Systolic Volume

FPG Fasting Plasma Glucose

FS Fractional Shortening

Hb Hemoglobin Concentration

HCT Hematocrit

HDL-C High-Density Lipoprotein Cholesterol

IVSd Interventricular Septal End-Diastolic Thickness

LA-ap Left Atrial Anteroposterior Diameter

LDL-C Low-Density Lipoprotein Cholesterol

LVEDD Left Ventricular End-Diastolic Diameter

LVESD Left Ventricular End-Systolic Diameter

LVPWd Left Ventricular Posterior Wall End-Diastolic Thickness

MCV Mean Corpuscular Volume

MPA Main Pulmonary Artery Diameter

MV A Mitral Valve Late Diastolic Flow Velocity

MV E Mitral Valve Early Diastolic Flow Velocity

PCI Percutaneous Coronary Intervention

PV Pulmonary Valve Blood Flow Velocity

RBC Red Blood Cell

RHR Resting Heart Rate

SV Stroke Volume

TC Total Cholesterol

TG Triglycerides

VO2 max Maximal Oxygen Uptake

VO2 peak Peak Oxygen Uptake

**References:**

1. Supriami K, Urbut SM, Tello-Ayala JR, Unlu O, Friedman SF, Abou-Karam R, et al. Genomic drivers of coronary artery disease and risk of future outcomes after coronary angiography. JAMA Netw Open. 2025;8:e2455368. https://doi.org/10.1001/jamanetworkopen.2024.55368.

2. Kanenawa K, Yamaji K, Kohsaka S, Ishii H, Amano T, Ando K, et al. Age‐stratified prevalence and relative prognostic significance of traditional atherosclerotic risk factors: a report from the nationwide registry of percutaneous coronary interventions in Japan. J Am Heart Assoc. 2023;12:e030881. https://doi.org/10.1161/JAHA.123.030881.

3. Du M, Ye X, Li D, Yang C, Dai R. Development of a prediction model for exercise tolerance decline in the exercise assessment of patients with acute myocardial infarction undergoing percutaneous coronary intervention revascularization in the acute phase. J Thorac Dis. 2023;15:4486–96. https://doi.org/10.21037/jtd-23-554.

4. Zhang Z, Wang F, Zhang Y, Yao J, Bi J, He J, et al. Associations of serum PFOA and PFOS levels with incident hypertension risk and change of blood pressure levels. Environ Res. 2022;212:113293. https://doi.org/10.1016/j.envres.2022.113293.

5. Jun-Ren Z, Run-Lin G, Shui-Ping Z, Guo-Ping L, Dong Z. 2016 Chinese guidelines for the management of dyslipidemia in adults. J Geriatr Cardiol. 2018;15:1–29.

6. American Diabetes Association. Diagnosis and Classification of Diabetes Mellitus. Diabetes Care. 2014;37 Supplement_1:S81–90. https://doi.org/10.2337/dc14-S081.

7. Appropriate body-mass index for asian populations and its implications for policy and intervention strategies. Lancet. 2004;363:157–63. https://doi.org/10.1016/S0140-6736(03)15268-3.

8. Sobieraj P, Leśniewski M, Sawicka A, Siński M, Lewandowski J. Agreement between resting heart rate measured by unattended automated office and office blood pressure measurement, ambulatory blood pressure monitoring, or electrocardiography. J Clin Hypertens. 2024;26:1402–10. https://doi.org/10.1111/jch.14892.

9. Muraru D, Badano LP, Peluso D, Dal Bianco L, Casablanca S, Kocabay G, et al. Comprehensive analysis of left ventricular geometry and function by three-dimensional echocardiography in healthy adults. J Am Soc Echocardiogr. 2013;26:618–28.
