# Supplementary material for: Development and validation of a machine learning model for post-PCI exercise intolerance in patients with coronary artery disease via electronic medical records
Source: Front Public Health. 2026 Feb 10;14:1751325. doi: 10.3389/fpubh.2026.1751325 (PMC12929426; doi:10.3389/fpubh.2026.1751325)
Supplement: Supplementary file 2 [file Data_Sheet_1.docx]

**Development and validation of a machine learning model for post-PCI exercise intolerance in patients with coronary artery disease via electronic medical records**

**List of Supplemental Table:**

Supplemental Table S1. Baseline clinical characteristics of patients in the training and testing data.

Supplemental Table S2. Summary of Key CPET Variables.

Supplemental Table S3. Results of the Shapiro–Wilk Normality Tests for Continuous Variables.

Supplemental Table S4. LASSO selection of variables and covariance analysis.

Supplemental Table S5. Tuning ranges and optimal hyperparameter values for each model.

Supplemental Table S6. AUC-ROC comparison of different ML models on the test dataset.

| **Table S1 Baseline clinical characteristics of patients in the training and testing data.** | | | | | |
| --- | --- | --- | --- | --- | --- |
| **Characteristics** | **Traing set (n = 402)** | **Testing set (n = 173)** | **Statistic** | ***P*** | **Missing (n,%)** |
| **Exercise Tolerance** |  |  | χ²=2.22 | 0.136 | 0 (0.0) |
| Exercise tolerance | 315 (78.95) | 129 (73.30) |  |  |  |
| Exercise intolerance | 84 (21.05) | 47 (26.70) |  |  |  |
| **Demographics and medical history** | | | | | |
| Age, years | 64.00 (56.00, 70.00) | 65.00 (57.75, 72.00) | Z=-1.53 | 0.126 | 0 (0.0) |
| Hight, cm | 165.00 (160.00, 170.00) | 165.00 (159.75, 170.00) | Z=-0.33 | 0.744 | 0 (0.0) |
| Weight, kg | 67.00 (59.50, 76.00) | 66.00 (60.00, 75.00) | Z=-0.27 | 0.783 | 0 (0.0) |
| BMI | 24.77 ± 3.48 | 24.97 ± 3.38 | t=-0.65 | 0.514 | 0 (0.0) |
| Gender |  |  | χ²=0.00 | 0.963 | 0 (0.0) |
| Male | 269 (67.42) | 119 (67.61) |  |  |  |
| Female | 130 (32.58) | 57 (32.39) |  |  |  |
| Type of CAD |  |  | χ²=0.75 | 0.388 | 0 (0.0) |
| SA | 241 (60.40) | 113 (64.20) |  |  |  |
| ACS | 158 (39.60) | 63 (35.80) |  |  |  |
| Smoking |  |  | χ²=1.65 | 0.199 | 0 (0.0) |
| No | 280 (70.18) | 114 (64.77) |  |  |  |
| Yes | 119 (29.82) | 62 (35.23) |  |  |  |
| Hypertension |  |  | χ²=1.08 | 0.299 | 0 (0.0) |
| No | 191 (47.87) | 76 (43.18) |  |  |  |
| Yes | 208 (52.13) | 100 (56.82) |  |  |  |
| Hyperlipidemia |  |  | χ²=0.02 | 0.890 | 0 (0.0) |
| No | 154 (38.60) | 69 (39.20) |  |  |  |
| Yes | 245 (61.40) | 107 (60.80) |  |  |  |
| Diabetes |  |  | χ²=0.36 | 0.548 | 0 (0.0) |
| No | 299 (74.94) | 136 (77.27) |  |  |  |
| Yes | 100 (25.06) | 40 (22.73) |  |  |  |
| CPET timing |  |  | χ²=5.06 | 0.080 | 0 (0.0) |
| <1 week | 50 (12.53) | 33 (18.75) |  |  |  |
| 1–3 weeks | 231 (57.89) | 87 (49.43) |  |  |  |
| 4–6 weeks | 118 (29.57) | 56 (31.82) |  |  |  |
| **Echocardiographic examination** | | | | | |
| LVEDD, mm | 47.00 (44.00, 50.00) | 47.00 (45.00, 50.00) | Z=-0.19 | 0.851 | 0 (0.0) |
| LVESD, mm | 29.00 (27.00, 32.00) | 29.00 (27.00, 32.00) | Z=-0.26 | 0.797 | 0 (0.0) |
| IVSd, mm | 10.00 (9.00, 10.00) | 9.00 (8.00, 10.00) | Z=-1.19 | 0.235 | 0 (0.0) |
| LVPWd, mm | 9.00 (9.00, 10.00) | 9.00 (8.00, 10.00) | Z=-0.97 | 0.332 | 0 (0.0) |
| LA-ap, mm | 34.00 (31.00, 37.00) | 34.00 (31.00, 37.00) | Z=-0.21 | 0.837 | 0 (0.0) |
| AO-a, mm | 21.00 (19.00, 22.00) | 20.50 (19.00, 22.00) | Z=-0.81 | 0.418 | 0 (0.0) |
| AO-s, mm | 33.00 (31.00, 36.00) | 33.00 (31.00, 36.00) | Z=-0.80 | 0.425 | 0 (0.0) |
| AO-asc, mm | 33.00 (31.00, 35.00) | 33.00 (30.00, 35.00) | Z=-0.43 | 0.664 | 0 (0.0) |
| MPA, mm | 22.00 (21.00, 24.00) | 22.00 (21.00, 23.00) | Z=-0.42 | 0.678 | 0 (0.0) |
| EDV, mL | 102.00 (88.00, 118.00) | 102.00 (92.00, 118.00) | Z=-0.30 | 0.762 | 0 (0.0) |
| ESV, mL | 34.00 (28.00, 42.00) | 34.00 (27.75, 41.00) | Z=-0.57 | 0.568 | 0 (0.0) |
| EF, % | 67.00 (62.00, 71.00) | 67.00 (62.00, 72.00) | Z=-0.67 | 0.501 | 0 (0.0) |
| FS, % | 38.00 (34.00, 40.00) | 37.00 (34.00, 41.00) | Z=-0.50 | 0.614 | 0 (0.0) |
| SV, mL | 68.00 (59.00, 77.00) | 67.00 (57.75, 77.00) | Z=-0.03 | 0.979 | 0 (0.0) |
| CO, mL/min | 4830.00 (4063.50, 5613.00) | 4842.00 (4242.25, 5775.25) | Z=-0.64 | 0.521 | 24 (4.1) |
| CI, mL/min/m² | 2819.00 (2418.00, 3232.50) | 2854.50 (2447.25, 3210.75) | Z=-0.45 | 0.651 | 24 (4.1) |
| MV E, m/s | 0.70 (0.60, 0.80) | 0.70 (0.60, 0.80) | Z=-0.85 | 0.398 | 0 |
| MV A, m/s | 0.80 (0.70, 0.90) | 0.80 (0.70, 1.00) | Z=-2.64 | 0.008 | 0 |
| E/A | 0.86 (0.71, 1.12) | 0.83 (0.70, 1.00) | Z=-1.61 | 0.107 | 0 |
| AV, m/s | 1.20 (1.10, 1.40) | 1.20 (1.10, 1.40) | Z=-1.23 | 0.218 | 0 |
| PV, m/s | 0.90 (0.80, 1.00) | 0.90 (0.80, 1.00) | Z=-1.36 | 0.174 | 8 (1.3) |
| e's, cm/s | 7.00 (5.00, 8.00) | 6.00 (5.00, 8.00) | Z=-0.50 | 0.615 | 8 (1.3) |
| e'l, cm/s | 9.00 (7.00, 11.00) | 9.00 (7.00, 10.00) | Z=-0.45 | 0.652 | 15 (2.6) |
| E/e | 9.00 (7.00, 11.00) | 9.00 (7.75, 11.00) | Z=-0.73 | 0.467 | 15 (2.6) |
| **Laboratory testing** | | | | | |
| RHR, bpm | 74.00 (69.00, 82.00) | 75.00 (69.75, 85.00) | Z=-1.14 | 0.254 | 0 (0.0) |
| RBC, 10^12/L | 4.68 (4.36, 4.97) | 4.59 (4.20, 4.96) | Z=-1.54 | 0.124 | 0 (0.0) |
| Hb, g/L | 141.00 (132.00, 151.00) | 142.00 (130.75, 150.00) | Z=-0.08 | 0.935 | 0 (0.0) |
| HCT, % | 41.50 (38.40, 44.05) | 41.15 (38.30, 43.90) | Z=-0.68 | 0.495 | 0 (0.0) |
| MCV, fL | 88.40 (86.00, 91.20) | 89.35 (86.80, 92.30) | Z=-2.73 | 0.006 | 0 (0.0) |
| TG, mmol/L | 1.39 (1.00, 2.08) | 1.28 (0.93, 2.11) | Z=-1.05 | 0.292 | 0 (0.0) |
| TC, mmol/L | 4.70 (3.77, 5.69) | 4.98 (3.75, 5.90) | Z=-0.97 | 0.333 | 0 (0.0) |
| LDL-C, mmol/L | 2.91 (2.13, 3.67) | 3.05 (2.06, 3.82) | Z=-0.60 | 0.552 | 0 (0.0) |
| HDL-C, mmol/L | 1.30 (1.10, 1.58) | 1.27 (1.11, 1.56) | Z=-0.02 | 0.987 | 0 (0.0) |
| CK, U/L | 97.00 (67.50, 135.00) | 89.50 (65.00, 117.50) | Z=-1.88 | 0.061 | 0 (0.0) |
| Exercise intolerance was defined as VO₂peak <16 mL·kg⁻¹·min⁻¹. Continuous variables are expressed as mean ± SD or median (IQR), as appropriate; categorical variables are shown as n (%). t: t test, Z: Mann–Whitney U test, χ²: Chi-square test; P values <0.05 were considered statistically significant and are highlighted in red; Reference ranges: LVEDD 42–58 mm (men), 38–52 mm (women); LVESD 25–40 mm (men), 22–35 mm (women); IVSd 6–10 mm (men), 6–9 mm (women); LVPWd 6–10 mm (men), 6–9 mm (women); LA-ap ≤40 mm; aortic sinus 29–39 mm (men), 27–35 mm (women); ascending aorta 26–36 mm (men), 23–33 mm (women); EDV 67–155 mL (men), 46–106 mL (women); ESV 22–58 mL (men), 14–42 mL (women); EF 55–70%; FS 28–44%; SV 60–100 mL; CO 4–8 L/min; CI 2.5–4.0 L/min/m²; E/A ≈1.0–2.0 (age dependent); AV ≤2.0 m/s; PV 0.6–0.9 m/s; e′s ≥7 cm/s; e′l ≥10 cm/s; E/e <14; RHR 60–100 bpm; RBC 4.2–5.9×10¹²/L (men), 3.9–5.0×10¹²/L (women); Hb 130–170 g/L (men), 120–150 g/L (women); HCT 40–50% (men), 36–44% (women); MCV 80–100 fL; TG <1.7 mmol/L; TC <5.2 mmol/L; LDL-C <3.4 mmol/L; HDL-C ≥1.0 mmol/L (men), ≥1.3 mmol/L (women); FBG 3.9–6.1 mmol/L; CK 40–200 U/L (men), 26–190 U/L (women). | | | | | |

| **Table S2. Summary of Key CPET Variables** | | | | | |
| --- | --- | --- | --- | --- | --- |
| **Statistic** | **VO2peak** | **AT** | **ATHR** | **HRmax** | **VE/VCO2 slope** |
| N | 575 | 575 | 575 | 575 | 575 |
| Mean | 19.29 | 14.09 | 110.03 | 129.13 | 31.89 |
| SD | 4.41 | 3.21 | 15.95 | 19.45 | 4.92 |
| Min | 9.9 | 7.3 | 17 | 71 | 20.28 |
| Max | 36.6 | 28.1 | 168 | 184 | 57.71 |
| Median | 18.9 | 13.5 | 110 | 129 | 30.94 |
| P25 | 16.3 | 12 | 99 | 115 | 28.7 |
| P75 | 21.75 | 15.6 | 120 | 142 | 34.53 |
| IQR | 5.45 | 3.6 | 21 | 27 | 5.84 |
| Abbreviations: VO₂ peak, peak oxygen uptake (mL·kg⁻¹·min⁻¹); AT, anaerobic threshold (mL·kg⁻¹·min⁻¹); ATHR, anaerobic threshold heart rate (beats/min); HRmax, maximum heart rate (beats/min); VE/VCO₂ slope, ventilatory equivalent for carbon dioxide slope (unitless). | | | | | |

| **Table S3. Results of the Shapiro–Wilk Normality Tests for Continuous Variables** | | |
| --- | --- | --- |
| **Sample** | **Statistic** | ***P value*** |
| Age | 0.992 | 0.004 |
| Height | 0.992 | 0.003 |
| Weight | 0.991 | 0.001 |
| BMI | 0.997 | 0.399 |
| LVEDD | 0.989 | <.001 |
| LVESD | 0.938 | <.001 |
| IVSd | 0.933 | <.001 |
| LVPWd | 0.408 | <.001 |
| LA-ap | 0.950 | <.001 |
| AO-a | 0.121 | <.001 |
| AO-s | 0.942 | <.001 |
| AO-asc | 0.983 | <.001 |
| MPA | 0.963 | <.001 |
| EDV | 0.973 | <.001 |
| ESV | 0.884 | <.001 |
| EF | 0.148 | <.001 |
| FS | 0.974 | <.001 |
| SV | 0.991 | 0.002 |
| CO | 0.985 | <.001 |
| CI | 0.980 | <.001 |
| MV E | 0.886 | <.001 |
| MV A | 0.971 | <.001 |
| E/A | 0.890 | <.001 |
| AV | 0.384 | <.001 |
| PV | 0.590 | <.001 |
| e's | 0.956 | <.001 |
| e'l | 0.979 | <.001 |
| E/e | 0.841 | <.001 |
| RHR | 0.893 | <.001 |
| RBC | 0.985 | <.001 |
| Hb | 0.984 | <.001 |
| HCT | 0.942 | <.001 |
| MCV | 0.685 | <.001 |
| TG | 0.418 | <.001 |
| TC | 0.995 | 0.04 |
| LDL-C | 0.426 | <.001 |
| HDL-C | 0.059 | <.001 |
| CK | 0.772 | <.001 |
| Shapiro–Wilk test statistic and P value for each continuous variable. P < 0.05 indicates non-normal distribution. | | |

| **Table S4. LASSO selection of variables and covariance analysis** | | |
| --- | --- | --- |
| **Variables** | **Coefficient of LASSO** | **VIF** |
| Age | 0.052 | 1.206 |
| BMI | 0.106 | 1.103 |
| Diabetes | 0.597 | 1.075 |
| Gender | 0.642 | 1.573 |
| Hb | -0.019 | 3.106 |
| RBC | -0.467 | 2.904 |
| RHR | 0.036 | 1.107 |
| Smoking | 0.946 | 1.269 |
| VIF < 5 is considered to be non-multicollinear. Abbreviations: LASSO, least absolute shrinkage and selection operator; VIF, variance inflation factor; BMI, Body mass index; Hb, glycated hemoglobin A1c; RBC, red blood cell count; RHR, resting heart rate. | | |

| **Table S5**. Tuning ranges and optimal hyperparameter values for each model. | | | |
| --- | --- | --- | --- |
| Model | Hyperparameter | Tuning range | Optimal value |
| LR | n jobs | - | -1 |
|  | alpha | list(range(0,1,0.05) | 0.1 |
|  | lambda | 10^list(range(log10(0.5),log10(0.001),length.out=50) | 3.77081389 |
| RF | ntree | list(range(100, 1000, 50) | 900 |
|  | mtry | list(range(1,13,1) | 3 |
|  | nodesize | list(range(2,10,1) | 5 |
|  | class weight | - | balanced |
| SVM | C | 2^seq(log2(0.1), log2(40), by=0.5) | 6.4 |
|  | kernal | - | rbf |
| KNN | k | list(range(10,200,by =5) | 95 |
|  | distance | c(1,2) | 2 |
|  | kernal | uniform, distance | distance |
| XGB | n estimators | list(range(100, 1000, 50) | 350 |
|  | learning rate | list(range(0.01,0.4,by=0.05) | 0.06 |
|  | max depth | list(range(2,10,by=1) | 8 |
|  | min child weight | c(1,2,3,4,5,6) | 5 |
|  | gamma | c(0, 0.2, 0.5, 1, 3) | 3 |
|  | subsample | list(range(0.05,1,by=0.05) | 0.7 |
|  | colsample bytree | list(range(0.2,1.0,by=0.2) | 0.4 |
|  | reg lambda | list(range(0,10,by=1) | 4 |
|  | booster | - | gbtree |
| MLP | hiden layzer sizes | list(c(10),c(20),c(30),c(60),c(16,16),c(24,12),c(32,16),c(32,32),c(64,64),c(128,128)) | 20 |
|  | activation | relu,tanh,logistic,identity | relu |
|  | learning rate | c(0.001,0.005,0.01,0.05,0.1) | 0.001 |
|  | max iter | - | 1000 |
|  | alpha | c(1e-5, 1e-4, 1e-3) | 1e-5 |
| LightGBM | num leaves | c(2,3,4) | 2 |
|  | max depth | c(4,8,16) | 4 |
|  | min child samples | c(15,20,25,30,35,40) | 35 |
|  | learning rate | list(range(0.05,0.2,by=0.01) | 0.12 |
|  | n estmators | list(range(100, 1000, 50) | 200 |
|  | reg alpha | c(0,0.1,0.5,1) | 0.1 |
|  | reg lambda | c(0,0.1,0.5,1) | 1 |
|  | feature fraction | c(0.4,0.5,0.6,0.7,0.8,0.9) | 0.4 |
|  | bagging fraction | c(0.6,0.8,0.9) | 0.9 |
|  | bagging feq | - | 1 |
|  | class weight | - | balanced |
| "–" denotes that the parameter was fixed (default or manually set) rather than tuned; the listed value represents its setting in the final model. The optimal hyperparameters for each model were determined by maximizing the F1 score within the cross-validation framework. | | | |

| **Table S6. AUC-ROC comparison of different ML models on the test dataset** | | | | | | | |
| --- | --- | --- | --- | --- | --- | --- | --- |
| **Models** | **LR** | **RF** | **SVM** | **KNN** | **XGB** | **MLP** | **Light GBM** |
| LR | - | 0.469 | 0.596 | 0.250 | 0.177 | 0.848 | 0.245 |
| RF | 0.469 | - | 0.811 | 0.738 | 0.550 | 0.585 | 0.685 |
| SVM | 0.596 | 0.811 | - | 0.536 | 0.384 | 0.737 | 0.501 |
| KNN | 0.250 | 0.738 | 0.536 | - | 0.762 | 0.341 | 0.925 |
| XGB | 0.177 | 0.550 | 0.384 | 0.762 | - | 0.241 | 0.841 |
| MLP | 0.848 | 0.585 | 0.737 | 0.341 | 0.241 | - | 0.327 |
| Light GBM | 0.245 | 0.685 | 0.501 | 0.925 | 0.841 | 0.327 | - |
